# Erratum und republication: Accreditation and qualification of primary care teaching practices in Germany – a nationwide online survey of universities

**DOI:** 10.3205/zma001849

**Published:** 2026-04-15

**Authors:** Sabine Gehrke-Beck, Isabel Kitte, Irmgard Streitlein-Böhme, Tobias Deutsch, Iris Demmer, Maryna Gornostayeva, Ralf Jendyk

**Affiliations:** 1 Charité – Universitätsmedizin Berlin, corporate member of Freie Universität Berlin and Humboldt-Universität zu Berlin, Institut für Allgemeinmedizin, Berlin, Germany; 2 Charité – Universitätsmedizin Berlin, Ambulantes Gesundheitszentrum AGZ, Berlin, Germany; 3 Medizinische Hochschule Hannover, Institut für Allgemeinmedizin und Palliativmedizin, Hannover, Germany; 4 Gesellschaft für Hochschullehre in der Allgemeinmedizin, Berlin, Germany; 5 Ruhr-Universität Bochum, Medizinische Fakultät, Abteilung für Allgemeinmedizin, Bochum, Germany; 6 Universität Leipzig, Medizinische Fakultät, Institut für Allgemeinmedizin, Leipzig, Germany; 7 Universitätsmedizin Göttingen, Medizinisches Versorgungszentrum, Göttingen, Germany; 8 Medizinische Fakultät Mannheim der Universität Heidelberg, Zentrum für Präventivmedizin und Digitale Gesundheit (CPD), Abteilung Allgemeinmedizin, Mannheim, Germany; 9 Deutsche Gesellschaft für Allgemeinmedizin (DEGAM), Sektion Studium und Hochschule, Berlin, Germany; 10 Universitätsmedizin Rostock, Institut für Allgemeinmedizin, Rostock, Germany

Erratum for: Gehrke-Beck S, Kitte I, Streitlein-Böhme I, Deutsch T, Demmer I, Gornostayeva M, Jendyk R. Accreditation and qualification of primary care teaching practices in Germany – a nationwide online survey of universities. GMS J Med Educ. 2025;42(5):Doc56. DOI: 10.3205/zma001780

## Notification

After publication of the work

Gehrke-Beck S, Kitte I, Streitlein-Böhme I, Deutsch T, Demmer I, Gornostayeva M, Jendyk R. Accreditation and qualification of primary care teaching practices in Germany – a nationwide online survey of universities. GMS J Med Educ. 2025;42(5):Doc56. DOI: 10.3205/zma001780

the authors have discovered that an incorrect reference list had been included during the editing process of the article. 

Therefore the article has been corrected and republished: 

Gehrke-Beck S, Kitte I, Streitlein-Böhme I, Deutsch T, Demmer I, Gornostayeva M, Jendyk R. Accreditation and qualification of primary care teaching practices in Germany – a nationwide online survey of universities. GMS J Med Educ. 2026;43(4):Doc55. DOI: 10.3205/zma001848

In the corrected version, the bibliography has been replaced, otherwise the article has remained unchanged. 

## Authors’ ORCIDS

- Sabine Gehrke-Beck: [0000-0002-6221-2813]
- Iris Demmer: [0000-0001-9652-9803]
- Ralf Jendyk: [0000-0002-2776-0515]

## Competing interests

The authors declare that they have no competing interests. 

